# Pruning System and Foliar Application of MgSO_*4*_ Alter Yield and Secondary Metabolite Profile of *Rosa damascena* under Rainfed Acidic Conditions

**DOI:** 10.3389/fpls.2017.00507

**Published:** 2017-04-12

**Authors:** Probir K. Pal, Mitali Mahajan

**Affiliations:** ^1^Agrotechnology of Medicinal, Aromatic and Commercially Important Crops, Council of Scientific and Industrial Research – Institute of Himalayan Bioresource TechnologyPalampur, India; ^2^Academy of Scientific and Innovative Research, Council of Scientific and Industrial Research – Institute of Himalayan Bioresource TechnologyPalampur, India

**Keywords:** partial pruning, MgSO_4_, rainfed acidic conditions, secondary metabolite, monoterpenoids, hydrocarbons, essential oil

## Abstract

Damask rose (*Rosa damascena* Mill.) is one of the most high-value essential oil-bearing plants in the world. However, the flower yield and quality of essential oil of *R. damascena* are largely influenced by the pruning practices and balanced supply of plant nutrition. The objective of this study was to test the hypothesis whether the pruning system and foliar fertilization of MgSO_4_ would influence the flower yield, growth and secondary metabolites profile of *R. damascena*. A field experiment of 10 treatment combinations comprising two pruning systems (complete and partial) and five levels of MgSO_4_ (water spray, MgSO_4_ @ 5.0g L^-1^, 10.0g L^-1^,15.0g L^-1^, and 20.0g L^-1^) was conducted. The experiment was conducted in randomized block design with factorial arrangement. Overall, the flower yield ranged from 503.66 to 1114.47 g bush^-1^, while oil content varied from 0.039 to 0.046% of the fresh flower. Irrespective of foliar spray, partial pruning produced significantly (*P* ≤ 0.05) higher flower yield (893.02 and 503.66 g bush^-1^) compared with complete pruning system in both the years. Regardless of pruning system, the foliar application of MgSO_4_ @ 15.0g L^-1^ registered about 26–38% higher flower yield compared with water spray control. The major constituents of essential oil were citronellol (19.75–48.88%), E-geraniol (9.63–29.6%), Z-citral (0.07–5.97%), nonadecane (6.76–22.32%), and heneicosane (2.87–10.21%). The principal component analysis revealed that the major hydrocarbons such as nonadecene, nonadecane, and heptadecane are positively and highly correlated with each others. The results suggest that higher yield and quality of *R. damascena* can be achieved through partial pruning system in combination with foliar application MgSO_4_ under rainfed acidic conditions.

## Introduction

Damask rose (*Rosa damascena* Mill.), a perennial shrub of the Rosaceae family, is widely known for its high-value essential oil content in the flower. Though *R. damascena* is being commercially cultivated in different parts of the world (Tabaei-Aghdaei et al., 2006; Pal, 2013), Bulgaria and Turkey are the main producers of rose essential oil in the World market (Rusanov et al., 2005, 2009). Among the 200 species of the genus *Rosa, R. damascena* is recognized as the most superior for high-value essential oil, which is extensively used in the flavoring and fragrance industries (Lawrence, 1991; Rusanov et al., 2009). Besides its application in aromatic industries, some pharmacological effects such as antioxidant, antibacterial and antimicrobial of rose essential oil have been reported (Ardogan et al., 2002; Achuthan et al., 2003; Basim and Basim, 2003; Ozkan et al., 2004; Kheirabadi et al., 2008; Rakhshandeh et al., 2008).

Although *R. damascena* is adapted to a wide range of environmental conditions, the quality of essential oil is mainly controlled by the genotype, time of flower harvesting, harvesting stage, distillation methods, and agronomic factors (Baydar and Baydar, 2005; Shawl and Adams, 2009). However, the relative proportions of the major components in the rose oil are the key factor to determine the quality of oil. Because of the low oil content in flower and lack of synthetic substitutes, rose oil is the most expensive essential oil compared with other essential oil in the world markets.

It has also been reported that the flower yield of *R. damascena* is considerably influenced by the crop-ecology and agronomic practices (Pal and Singh, 2013). Pruning is one of the most important agronomic practices for different rose species to increase flower size, quality and color of flower (Gibson, 1984; Anderson, 1991). Pruning operation modifies the growth phases and physiological activities for facilitating new axillary bud initiation. In pruned stems, the flower initiation starts shortly after axillary bud development (Chimonidou et al., 2000). Moreover, pruning operation is carried out to improve the shape of the plant for facilitating cultural operations and harvesting. It has also been reported that the pruning operation promotes photosynthetic light reaction, increases metabolic sinks, and elevates turgor pressure in plants (Calatayud et al., 2007). The pruning style also influences the nutrient cycle (Admasu and Struikb, 2000). However, roses need different types, level and timing of pruning depending upon their species, variety, and ecological conditions (Hessayon, 1988; Pal et al., 2014). Thus, there is a pressing need to standardize the pruning system to maintain the rose bushes in a manageable condition for plucking the flower and enhancing production under different agro-climatic conditions.

In Palampur (western Himalayan region), the rainfall is erratic, and 70–80% of the total rain is received during monsoon (June–September); but the gentle winter rain is useful for crop productions. Now there is a pressing need for enhancing crop productivity under rainfed conditions. However, the efficiency of plant nutrients applied in the soil is low under rainfed conditions. Moreover, unpredicted rainfall also makes difficult to determine the level and timing of fertilizer application.

The nutritional factor is equally responsible for determining the flower yield and quality of essential oil of *R damascena*. Among the essential plant nutrients, magnesium (Mg^2+^) is one of the important secondary nutrients, which occurs in the center of the chlorophyll molecule and therefore plays a major role in plant photosynthesis (Ding et al., 2008; Hermans et al., 2010). It has also been reported that even minute differences in Mg may influence the various chloroplast enzymes (Shaul, 2002). Nevertheless, the uptake of Mg^2+^ by plant is lower than K due to cationic competitive effects. Moreover, the Mg depletion in soil is a growing concern for intensive agriculture, particularly when soil fertilized only with N, P, and K. Mg deficiency is a more serious problem in rainfed acidic soil conditions due to the interaction with aluminum (Al). Thus, nutrient use efficiency (NUE) is very low under this situation. Furthermore, under rainfed conditions, application of nutrient in soil cannot meet the internal demand during critical stages. The foliar application of plant nutrients is an alternative approach to increase the NUE and to meet the internal demand under these conditions. Under nutrient deficiency condition, the foliar application technique ensures instant translocation of nutrients to various plant parts through leaf tissues (Fageria et al., 2009). However, the effects of foliar application of Mg and their amount on *R. damascena* have not been studied lucidly under rainfed acidic conditions. The objectives of this study were to investigate the impact of pruning system and foliar application of different concentrations of Mg^2+^ on the flower yield, essential oil content, and composition of essential oil of *R. damascena* under rainfed acidic soil conditions.

## Materials and Methods

### Experimental Location, Climate, and Soil Characteristics

**FIGURE 1:**
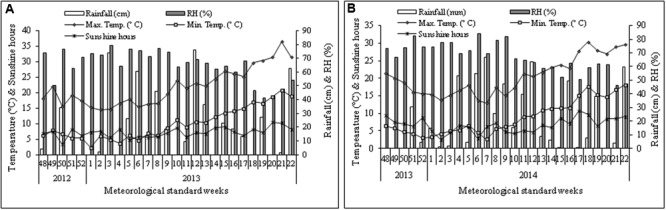
**Weekly mean maximum and minimum temperature (°C), sunshine hours (SS), rainfall (cm), and relative humidity (RH %) during the cropping season of 2012–2013 (A)** and 2013–2014 **(B)** at Palampur, India. The starting date of 48th meteorological standard week (MSW) and closing date of 22nd MSW are 26th November and 3rd June, respectively.

The field experiment was conducted at the experimental farm of CSIR-Institute of Himalayan Bioresource Technology (32°06′05″N; 76°34′10″E), Palampur, India, during the cropping seasons of 2012–2013 and 2013–2014. According to the USDA soil taxonomy classification system, the soil of experimental area belongs to Alfisols (Sharma and Kumar, 2003). The experimental unit is situated at the altitude of 1393 m from mean sea-level. The amount and distribution of rainfall, maximum and minimum temperature, relative humidity, and sunshine hours during the two growing seasons were also presented (**Figure 1**). The soil of experimental plot was silty clay in texture, and the reaction of the soil was acidic. The physico-chemical properties of the experimental soil are presented in **Table 1**.

**Table 1:** Physico-chemical properties of the soil.

Property	Value
Soil texture	Silty clay
Sand (%)	11.2
Silt (%)	41.4
Clay (%)	47.4
pH (1:2)	5.53
Organic carbon (%)	1.11
Available nitrogen (kg ha^-1^)	281.47
Available phosphorus (kg ha^-1^)	11.28
Available potassium (kg ha^-1^)	782.30
Available calcium (kg ha^-1^)	62.90
Available magnesium (kg ha^-1^)	107.11
Available sulfur (kg ha^-1^)	38.41
Iron (ppm)	56.81
Manganese (ppm)	38.39
Zinc (ppm)	3.05
Copper (ppm)	1.07

### Plant Material, Crop Management, and Application of Treatments

In this study, 5-year-old plantation of *R. damascena* (*cv.* Jwala) field was used, and the planting geometry was 1.5 m between rows and 0.75 m within rows. A basal dose of 100 kg nitrogen (N), 21.85 kg phosphorus (P), and 41.50 kg potassium (K) was applied by urea (46% N), single super phosphate (16% P_2_O_5_), and muriate of potash (60% K_2_O), respectively, during both the cropping seasons. Irrigation was not given during the course of study, since the crop was grown under rain-fed conditions. However, other recommended agronomic practices for *R. damascena* were adopted as per requirement for better growth and development. The experiment was laid out in randomized block design (RBD) with two-factors factorial arrangement and three replications. Ten treatment combinations consisting two different types of pruning [complete pruning (C) and partial pruning (P)] and five different concentrations of MgSO_4_ [water spray control (M_0_), MgSO_4_ @ 5.0g L^-1^ (M_1_), MgSO_4_ @ 10.0g L^-1^ (M_2_), MgSO_4_ @ 15.0g L^-1^ (M_3_), and MgSO_4_ @ 20.0g L^-1^ (M_4_)] were tested. The pruning operation was done during 50th meteorological standard week (MSW) at 90 cm height from the ground level in 2012–2013 and 2013–2014. In case of partial pruning system, five new shoots were left without pruning in each bush, and the remaining shoots were pruned at 90 cm height from the ground level. The MgSO_4_ solutions for different treatments were diluted with water (about 800 L ha^-1^), and sprayed twice; the first foliar spray was applied at axillary bud development stage, and the second spray was done at flower bud appearance stage.

### Growth and Yield Data

Two plants were randomly selected for each treatment from each replication, and the selected plants were tagged for growth and yield observation. After pruning, number of old shoots (No. bush^-1^) was recorded. New shoot initiation rate (No. old shoot^-1^) was also recorded. The data on number of flowers (No. shoot^-1^), flower weight (g flower^-1^), flower yield (g new shoot^-1^ and g bush^-1^) were recorded day-to-day basis from initial date of harvesting to end of the flowering. The flowers were harvested by manual picking in the morning (6:00–9:00 AM) to prevent the loss of volatile compounds from the flower.

### Extraction of Essential Oil

The essential oil was extracted from fresh flowers harvested separately from each plot. The oil was extracted by hydro-distillation for 4 h on a Clevenger-type apparatus using a 5.0 L distillation system. The flower and water ratio was 1:2 (w/v). The essential oil from each plot sample was measured, and the oil content (w/w) in flower was expressed as percentage on a fresh weight basis. The extracted essential oil was dehydrated by anhydrous Na_2_ SO_4_ (Merck) and collected in a glass vial. The sealed oil samples were stored in a dark place at 4°C until analysis.

### GC–MS Analysis and Compound Identification

The compounds of oil samples were identified by using a Shimadzu QP2010 GC-MS system (Shimadzu, Tokyo, Japan) attached with an AOC-5000 auto injector and a ZB-5 (SGE International, Ringwood, VIC, Australia) fused silica capillary column (30 m × 0.25 mm i.d., and film thickness 0.25 μm). The conditions for analysis were identical to those previously described (Pal et al., 2014). The retention indices (RI) for all volatile compounds were computed by using homologous series of n-alkanes (C8–C24). Then the constituents of essential oil were identified by comparing their RI and mass spectra with those of authentic samples and with those stored in the NIST-MS (National Institute of Standards and Technology-mass spectral) database (Stein, 2005).

### GC Analysis and Quantification

All the GC analyses of rose oil samples were carried out by a Shimadzu GC-2010 gas chromatograph (Shimadzu, Tokyo, Japan) equipped with flame ionization detector (FID) and a ZB-5 capillary column (30 m × 0.25 mm, fused silica, and film thickness 0.25 m). The operating conditions for analysis were identical to those previously described (Pal et al., 2014). The nitrogen gas was used as carrier with the velocity of 1.05 mL min^-1^. Then the individual compounds were quantified based on peak area percentage of the chromatogram.

### Determination of Chlorophyll (Chl) and NPK in Leaf

At the time of peak flowering stage, the leaves were collected from each experimental unit for estimation of Chl content. Chl was extracted from 200 mg fresh leaf tissue sample for each treatment in the solution of 80% acetone (v/v). The absorbance of the extracts at 645 and 663 nm was recorded with a spectrophotometer (model T 90 + UV/vis, PG Instrument Ltd.). Finally, the total Chl content (mg g^-1^ tissue) was calculated based on the absorbance values as per standard equations (Arnon, 1949).

On the other hand, the leaves were collected from each experimental unit at the end of the both cropping seasons for the estimation of N, P, and K content in the leaves. After drying, the leaf samples were prepared with a laboratory grinder having a sieve spacing of 0.7 mm. For N estimation, the samples were digested with concentrated H_2_SO_4_ and a catalyst mixture of potassium sulfate and copper sulfate (10:1). Then, Kel Plus nitrogen analyzer unit was used for estimation of total N content in the leaves. In case of P and K estimation, a mixture of concentrated H_2_SO_4_ and perchloric acid (5:1) was used for digestion. Then, a spectrophotometer (model T 90 + UV/vis, PG Instrument Ltd.) and a flame photometer (model BWB XP, BWB technologies UK Ltd., UK) were used for the estimation of total P and K, respectively, as per standard procedure (Prasad et al., 2006).

### Statistical Analysis

The growth and yield data obtained from *R. damascena* for consecutive 2 years were subjected to analysis of variance (ANOVA) to test the sole effect of pruning system and foliar application of MgSO_4_, and pruning system × MgSO_4_ interaction by using Statistica 7 software (Stat Soft Inc., Tulsa, OK, USA). In this experiment, a two-factor factorial RBD was used with three replications. Differences among the treatment means were assessed by the least significant difference (LSD) value at *P* = 0.05. Correlation matrix was conducted by using Statistica 7 software to investigate relationships between the yield and yield attributes. The regression equation between yield and MgSO_4_ doses was also developed using same software. However, the heat maps of chemical profiling of essential oil were prepared with the help of R data analysis software (version 3.1.3). Principal component analysis (PCA) was performed to evaluate the influences of treatment combinations on chemical profiling of essential oil as a bi-plot, and nature of variations among the treatment combinations was also projected. The factor loading values represent the correlations of each variable with the principal components (PCs).

## Results

### Growth and Yield Data

The analyzed data revealed that two main yield attributes of *R. damascena*, new shoot initiation rate (No. old shoot^-1^) and number of flowers (No. bush^-1^), were significantly (*P* ≤ 0.05) influenced by the system of pruning during both the years (**Table 2**). In this study, partial pruning system registered significantly (*P* ≤ 0.05) higher new shoot initiation rate (4.63 and 10.80 No. old shoot^-1^) and number of flowers (350.47 and 266.80 No. bush^-1^) compared with the complete system, irrespective of foliar application of MgSO_4_. However, number of petals (No. flower^-1^) and flower weight (g flower^-1^) were not influenced by the system of pruning during both the years, and these two parameters remained inconsistent over the years.

**Table 2:** Effect of pruning system and foliar application of MgSO_4_ on yield attributes and flower yield of *Rosa damascena* under rainfed acidic conditions of western Himalayan region.

Treatment	New shoot initiation rate (No. old shoot^-1^)		Petals (No. flower^-1^)		Weight (g flower^-1^)		Flower yield (g new shoot^-1^)		Flower (No. bush^-1^)		Flower yield (g bush^-1^)		Blind shoot (%)	
	2012–2013	2013–2014	2012–2013	2013–2014	2012–2013	2013–2014	2012–2013	2013–2014	2012–2013	2013–2014	2012–2013	2013–2014	2012–2013	2013–2014
**System of pruning (S)**														
Complete pruning (C)	2.63	6.95	28.40	30.73	3.12	3.35	17.91	5.57	286.00	150.53	893.02	503.66	15.87	24.73
Partial pruning (P)	4.63	10.80	27.50	30.63	3.14	3.31	12.66	9.45	350.47	266.80	1098.68	881.97	13.95	18.12
SEM±	0.17	0.55	0.70	0.90	0.04	0.04	0.87	0.53	8.69	9.78	28.63	29.76	1.12	1.72
CD (*P* = 0.05)	0.50	1.64	NS	NS	NS	NS	2.56	1.55	25.63	28.86	84.62	87.97	NS	5.10
**Foliar spray (M)**														
Water spray (M_0_)	3.43	8.30	29.25	30.08	3.19	3.27	13.29	5.75	275.00	183.83	881.39	601.17	16.06	22.39
MgSO_4_ @ 5.0g L^-1^(M_1_)	3.70	7.82	27.83	31.42	3.13	3.38	16.31	7.58	317.50	178.50	992.63	600.35	14.20	20.98
MgSO_4_ @ 10.0g L^-1^ (M_2_)	3.54	10.53	27.58	31.00	3.11	3.37	16.10	7.16	341.17	196.00	1060.72	663.72	14.15	20.71
MgSO_4_ @ 15.0g L^-1^ (M_3_)	3.79	7.86	27.75	30.67	3.13	3.34	16.61	9.87	357.50	250.67	1114.47	830.69	14.19	21.15
MgSO_4_ @ 20.0g L^-1^ (M_4_)	3.68	9.88	27.33	30.25	3.09	3.29	14.07	7.17	300.00	234.33	930.06	768.12	15.96	21.90
SEM±	1.61	0.87	1.11	1.42	0.06	0.06	1.37	0.83	13.73	15.47	45.28	47.07	1.77	2.73
CD (*P* = 0.05)	NS	NS	NS	NS	NS	NS	NS	2.46	40.52	45.62	133.80	139.09	NS	NS
SEM± for (S × M)	0.37	1.24	1.56	2.00	0.08	0.08	1.94	1.18	19.42	21.87	64.03	66.56	2.50	3.86
CD (*P* = 0.05) for (S × M)	NS	NS	NS	NS	NS	NS	NS	3.47	57.30	64.52	189.20	196.71	7.41	NS

*NS indicates the differences among treatment means are not significant.*

The effects of foliar application of MgSO_4_ on new shoot initiation rate (No. old shoot^-1^), number of petals (No. flower^-1^), and flower weight (g flower^-1^) were not significant (*P* ≥ 0.05). However, number of flowers (no. bush^-1^), the important yield component of *R. damascena*, was significantly (*P* ≤ 0.05) influenced by the foliar application of MgSO_4_; and maximum numbers of flowers (357.50 and 250.67 no. bush^-1^) were recorded with MgSO_4_ @ 15.0g L^-1^ (**Table 2**). Irrespective of system of pruning, the application of MgSO_4_ @ 15.0g L^-1^ registered about 30 and 36% higher number of flowers compared with water spray control during 2012–2013 and 2013–2014, respectively. The percentage of blind shoots was also significantly (*P* ≤ 0.05) affected by the system of pruning during 2013–2014 cropping season; however, the effect of foliar application of MgSO_4_ on blind shoot (%) was insignificant, least percentages of blind shoot (14.15 and 20.71%) were recorded with the foliar application of MgSO_4_ @ 10.0g L^-1^ during both the years. The interaction effects of pruning system and foliar application of MgSO_4_ on blind shoot were significant (*P* ≤ 0.05) during 2012–2013 (**Table 2**).

The analyzed data revealed that the effects of pruning system and foliar application of MgSO_4_ on the flower yield (g bush^-1^) of *R. damascena* were significant (*P* ≤ 0.05) in both the years (**Table 2**). Regardless of foliar application of MgSO_4_, the partial pruning system increased flower yield by about 23 and 75% compared with complete pruning system during 2012–2013 and 2013–2014, respectively. Among the foliar treatments of MgSO_4_, the application of MgSO_4_ @ 15.0g L^-1^ recorded significantly (*P* ≤ 0.05) higher flower yield (1114.47 and 830.69 g bush^-1^) compared with water spray control during both the years (**Table 2**). Though the effects of the foliar application of MgSO_4_ @ 5.0g L^-1^, MgSO_4_ @ 10.0g L^-1^, and MgSO_4_ @ 15.0g L^-1^ on flower yield were statistically at par (*P* ≤ 0.05) in 2012–2013; significantly (*P* ≤ 0.05) higher flower yield was recorded with MgSO_4_ @ 15.0g L^-1^ compared with MgSO_4_ @ 5.0g L^-1^ and MgSO_4_ @ 10.0g L^-1^ in 2013–2014. Regardless pruning system, the foliar application of MgSO_4_ @ 15.0g L^-1^ registered about 26 and 38% higher flower yield compared with water spray control in 2012–2013 and 2013–2014, respectively. The interaction effect between pruning system and foliar application of MgSO_4_ on the flower yield (g bush^-1^) was significant (*P* ≤ 0.05) in 2012–2013 cropping season.

### Regression and Correlation Analysis

**FIGURE 2:**
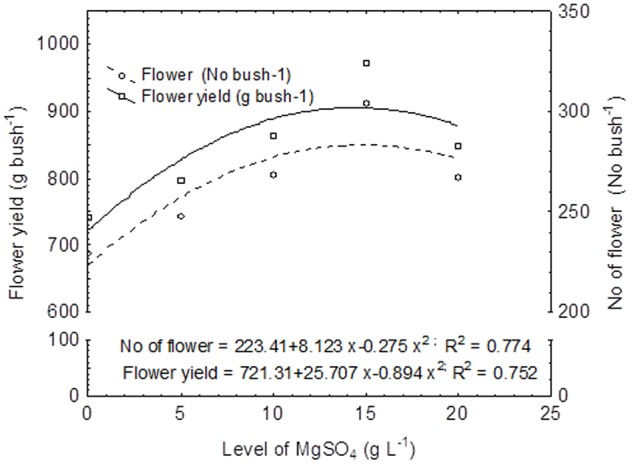
**Regression equation between foliar application of MgSO_4_ and yield (Number of flower and flower yield per bush).** The primary horizontal axis (X) represents different doses of MgSO_4_.

**FIGURE 3:**
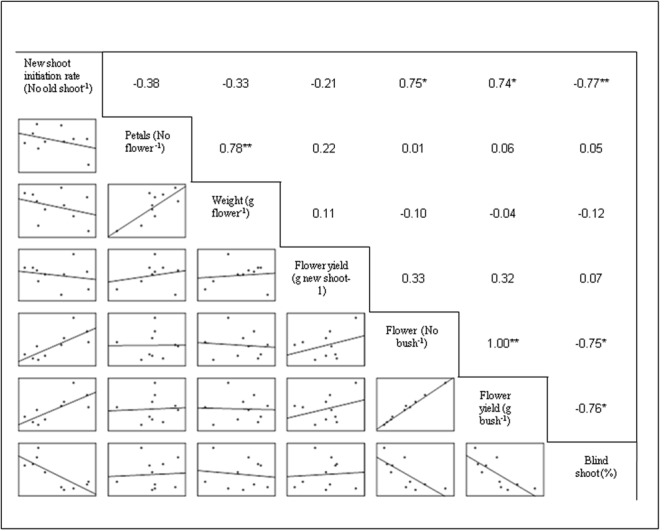
**Correlation matrix among new shoot initiation rate, yield, and yield components.** The mean values of the 2 years polled data of the corresponding treatments are used (where *N* = 10). ^∗^ and ^∗∗^ indicate that the corresponding values are significant at *P* ≤ 0.05 and *P* ≤ 0.01, respectively.

The regression equations were established considering MgSO_4_ doses as independent variable and flower yield and number of flowers as dependent variable (**Figure 2**). In this experiment, the flower yield was increased with the corresponding increasing concentration of MgSO_4_ doses up to 15.0 g L^-1^, thereafter the yield was declined. Thus, the second degree polynomial relationship (y = 721.31 + 25.707 x - 0.894 x^2^; *R*^2^ = 0.752) was found between flower yield and level of MgSO_4_ doses (**Figure 2**). The similar relation was found between number of flower and concentration of MgSO_4_ doses with an equation of y = 223.41 + 8.123 x - 0.275 x^2^, and *R*^2^ = 0.774. The correlation matrix among the flower yield, yield attributes, and growth parameter was also established, and the data revealed that flower yield (g bush^-1^) was significantly and positively correlated with the number of flower (*r* = 1.00; *P* ≤ 0.01) and new shoot initiation rate (*r* = 0.74; *P* ≤ 0.05) (**Figure 3**). However, a negative correlation was observed with the percentage of blind shoot (*r* = -0.76; *P* ≤ 0.05). The negative correlation (*r* = -0.77; *P* ≤ 0.01) was also found between the percentage of blind shoot and new shoot initiation rate. A significant (*P* ≤ 0.01) and a positive correlation (*r* = 0.78) was found between the flower weight (g flower^-1^) and number of petal (**Figure 3**).

### Oil Content (%)

**FIGURE 4:**
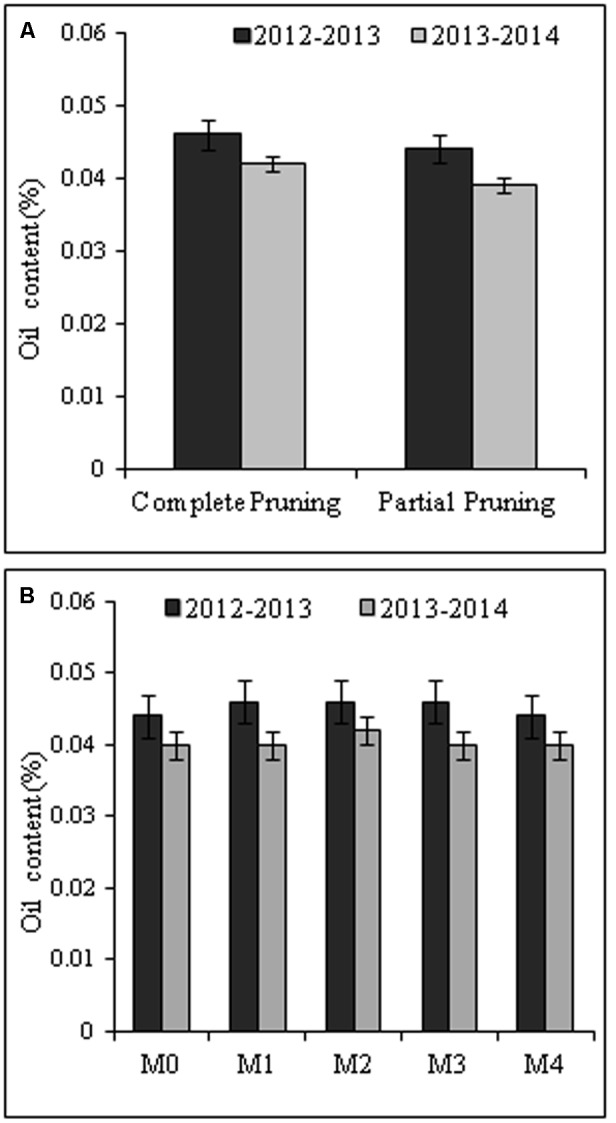
**The effect of pruning system (A)** and foliar application of MgSO_4_ **(B)** on essential oil content (%). The oil content in fresh flower was estimated based on oil recovery in laboratory scale. Vertical bars indicate mean standard error (±) at *P* = 0.05. M_0_, M_1_, M_2_, M_3_, and M_4_ are the level of MgSO_4_ @ 0.0, 5.0, 10.0, 15.0, and 20.0 g L^-1^ of water, respectively.

In our study there was no significant effect (*P* ≥ 0.05) of pruning system and foliar application of MgSO_4_ on the essential oil content (%) in the flowers of *R. damascena* during both the cropping seasons (**Figures 4A,B**). However, regardless of foliar spray of MgSO_4_, complete pruning system registered slightly higher oil content (0.046 and 0.042%) compared with the partial pruning system. Among the foliar treatments, the applications of MgSO_4_@ 5.0g L^-1^, @ 10.0g L^-1^, and @ 15.0g L^-1^ registered highest oil content (0.046%) in 2012–2013 cropping seasons irrespective of pruning system (**Figure 4B**). The lowest oil content (0.040 and 0.044%) was recorded with water spray control and higher doses of MgSO_4_ (@ 20.0g L^-1^) in both the years.

### Compositions of Essential Oil

**FIGURE 5:**
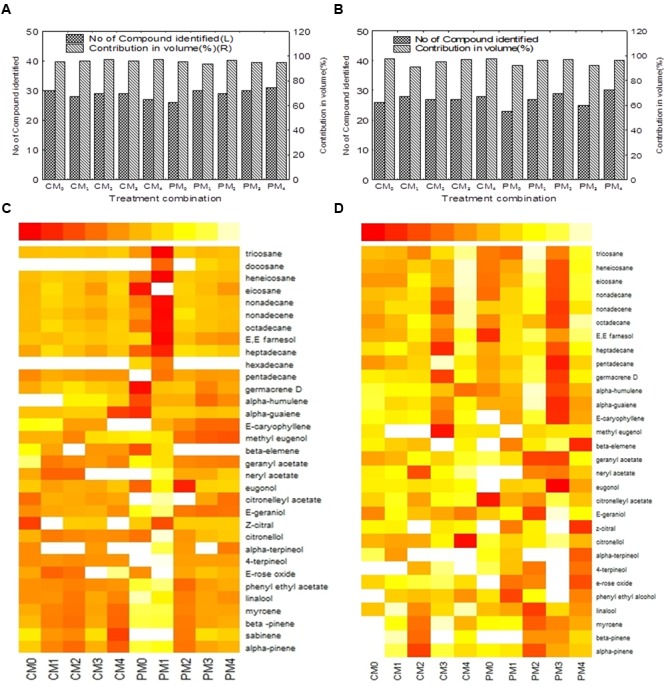
**Volatile compounds of essential oil after GC-MS analysis.** Total of 33 compounds were identified in 2012–2013, which contributed about 93–98% of total volume **(A)**; whereas 30 compounds were identified in 2013–2014 **(B)**. The heat map **(C,D)** representing dynamics of the volatile compounds as influenced by interaction effects of pruning system and foliar application of MgSO_4._ The left end of the heat map legend scale indicates maximum value. The changes of color from the left to right end of the heat map legend indicate decrease of the compound abundance. C and P are the complete and partial pruning, respectively, while M_0_, M_1_, M_2_, M_3_, and M_4_ are the level of MgSO_4_ @ 0.0, 5.0, 10.0, 15.0, and 20.0 g L^-1^ of water, respectively.

In this experiment, we have identified a total of 33 compounds in 2012–2013, which contributed about 93–98% of the total volume; whereas 30 compounds were identified in 2013–2014 (**Figures 5A,B**). The lowest numbers (26 and 23) of compounds were identified with the interaction effects of partial pruning system and water spray control treatment during both the years. However, the maximum contribution (97.25 and 97.54%) by the identified compounds in total volume of essential oil was observed with interactive effects of complete pruning system and foliar application of MgSO_4_ @ 20.0g L^-1^ and water spray.

The chemical profiles of rose essential oil under different treatment combinations are presented by heat maps (**Figures 5C,D**). The heat maps containing 33 and 30 compounds which depict the changes of chemical profiling of rose essential oil were observed due to pruning system and foliar application of MgSO_4_. The data in the heat map showed that the accumulation patterns of two major monoterpenoids, citronellol, and E-geraniol, were inconsistent over the years (**Figures 5C,D**). However, the maximum shearing of citronellol (39.82%) and E-geraniol (29.68%) was observed with PM_0_ (partial pruning with water spray control) and PM_3_ (partial pruning with foliar application of MgSO_4_ @ 15.0g L^-1^), respectively, in 2012–2013 cropping seasons (**Figure 5C**). In 2013–2014, CM_4_ (complete pruning with foliar application of MgSO_4_ @ 20.0g L^-1^) and PM_3_ (partial pruning with foliar application of MgSO_4_ @ 15.0g L^-1^) registered maximum quantity of citronellol (48.88%) and E-geraniol (24.84%), respectively (**Figure 5D**). Flowers obtained from the PM_2_ (partial pruning with MgSO_4_ @ 10.0g L^-1^) treatment registered highest concentration (1.27 and 1.45%) of linalool during both the years. In the present investigation, the two major hydrocarbons, nonadecane, and heneicosane, also show diverse accumulation patterns under different treatment combinations in both the years (**Figures 5C,D**). The minimal level of nonadecane accumulation was recorded with CM_1_ (complete pruning with foliar application of MgSO_4_ @ 5.0g L^-1^) followed by PM_2_ (partial pruning with foliar application of MgSO_4_ @ 10.0g L^-1^) in 2012–2013 season, whereas in second cropping season, the minimum shearing of nonadecane was recorded with higher concentration of MgSO_4_ @ 20.0g L^-1^) under both types of pruning system (**Figures 5C,D**).

### Total Chl, N, P, K, and Mg Concentration in Leaf

The results presented in the **Table 3** revealed that the effects of pruning system on the total Chl content in leaves were insignificant (*P* ≥ 0.05) during 2012–2013; however, partial pruning system registered significantly (*P* ≤ 0.05) higher Chl content (3.58 mg g^-1^) in 2013–2014. Although the effect of foliar application of MgSO_4_ on total Chl content in leaves was insignificant (*P* ≥ 0.05) in both the years, the Chl concentration was gradually increased with corresponding increasing concentration of MgSO_4_ and the utmost value (3.07 and 3.45 mg g^-1^) was attained with MgSO_4_ @ 15.0g L^-1^ during both the years. We also observed that the accumulation of N, P, and K in the leaf was significantly (*P* ≤ 0.05) influenced by pruning system, and the maximum values were registered with the partial pruning system during 2013–2014 cropping season. On the other hand, irrespective of pruning system, the effects of foliar application of MgSO_4_ on the accumulation of N and P were insignificant (*P* ≥ 0.05) in both the years. However, K concentration in leaf was significantly (*P* ≤ 0.05) influenced by the foliar application of MgSO_4_ in 2012–2013 cropping season, and the maximum value was recorded with water spray control. The Mg concentration in leaves was not influenced by the system of pruning during both the years. However, the effect of foliar application of MgSO_4_ on Mg accumulation in leaves was significant (*P* ≤ 0.05) during 2012–2013 cropping season and the maximum value was observed with MgSO4 @ 15.0g L^-1^. In case of interaction effect between pruning system and foliar application of MgSO_4_, the insignificant (*P* ≥ 0.05) results were found in both the cropping seasons (**Table 3**).

**Table 3:** Effect of pruning system and foliar application of MgSO_4_ on accumulation of total chlorophyll (Chl), nitrogen, phosphorus potassium, and magnesium in leaves of *R. damascena* grown under rainfed acidic conditions.

Treatment	Total Chl (mg g^-1^ fresh leaf tissue)		Nitrogen (mg g^-1^ dry leaf)		Phosphorus (mg g^-1^ dry leaf)		Potassium (mg g^-1^ dry leaf)		Magnesium (mg g^-1^ dry leaf)	
	2012–2013	2013–2014	2012–2013	2013–2014	2012–2013	2013–2014	2012–2013	2013–2014	2012–2013	2013–2014
**System of pruning (S)**										
Complete pruning (C)	2.84	3.16	21.17	19.91	4.02	2.57	11.73	8.63	2.85	2.60
Partial pruning (P)	3.05	3.58	21.19	22.37	4.28	3.35	11.17	9.75	2.83	2.51
SEM ±	0.08	0.09	0.52	0.36	0.08	0.13	0.20	0.26	0.07	0.06
CD (*P* = 0.05)	NS	0.25	NS	1.08	0.23	0.38	NS	0.77	NS	NS
**Foliar spray (M)**										
Water spray (M_0_)	2.77	3.14	20.05	20.68	3.91	2.68	11.85	9.17	2.49	2.46
MgSO4 @ 5.0g L^-1^ (M_1_)	2.92	3.27	20.63	20.75	4.07	2.94	11.84	9.17	2.80	2.54
MgSO4 @ 10.0g L^-1^ (M_2_)	3.06	3.39	21.77	21.22	4.12	2.95	11.69	9.18	2.99	2.55
MgSO4 @ 15.0g L^-1^ (M_3_)	3.07	3.45	22.32	21.45	4.29	3.29	10.12	8.83	3.01	2.63
MgSO4 @ 20.0g L^-1^ (M_4_)	2.91	3.42	21.13	21.6	4.37	2.93	11.79	9.59	2.90	2.59
SEM±	0.13	0.14	0.82	0.57	0.12	0.20	0.31	0.41	0.11	0.10
CD (*P* = 0.05)	NS	NS	NS	NS	NS	NS	0.93	NS	0.32	NS
SEm± for (S × M)	0.18	0.19	1.17	0.81	0.17	0.29	0.44	0.58	0.15	0.14
CD (*P* = 0.05) for (S × M)	NS	NS	NS	NS	NS	NS	NS	NS	NS	NS

*NS indicates the differences among treatment means are not significant.*

### Principal Component Analysis (PCA)

**FIGURE 6:**
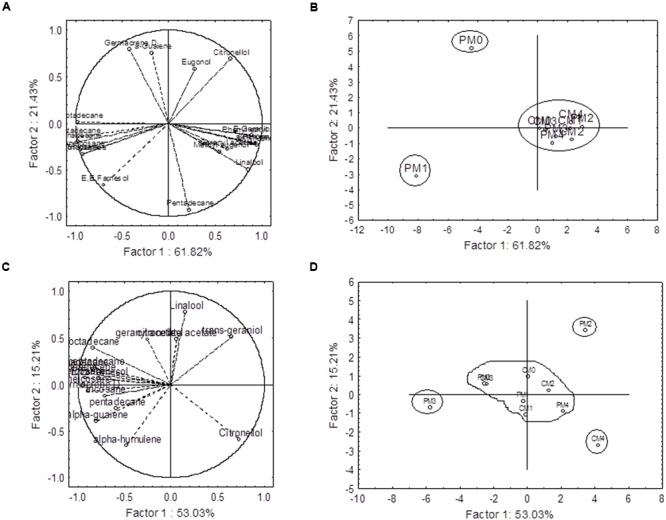
**Principal component analysis of secondary metabolites profiling data.** First component (PC_1_) and second component (PC_2_) jointly explained 83.25 and 68.24 % of the total variation in 2012–2013 **(A,B)** and 2013–2014 **(C,D)** cropping seasons, respectively. The projection of the variables (compounds) on the factor-plane (1 × 2) is presented in **(A,C)**. The factor loading values are presented as vectors in the space of the principal component analysis (PCA) bi-plots. C and P are the complete and partial pruning, respectively, while M_0_, M_1_, M_2_, M_3_, and M_4_ are the levels of MgSO_4_ @ 0.0, 5.0, 10.0, 15.0, and 20.0g L–^1^ of water, respectively.

The principal component analyses (PCA) were performed by using the sets of 20 and 17 compounds of essential oil for 2012–2013 and 2013–2014, respectively. The components, which are quantified in all the treatments, are used for PCA. The results from PCA revealed that the first component (PC_1_) and second component (PC_2_) jointly explained 83.25 and 68.24% of the total variations for 2012–2013 and 2013–2014 cropping seasons, respectively (**Figure 6**). The relationships among the variables in the space of the first two components (PC_1_ and PC_2_) with factor loadings are presented in **Figures 6A,C**, and indicate how each variable contributes to the PCs. In this experiment, PC_1_ has positive coefficients with α-pinene, β-pinene, myrcene, linalool, phenyl ethyl acetate, citronellol, E-geraniol, eugenol, geranyl, acetate, methyl eugenol, and α-guaiene in first cropping season (**Figure 6A**). However, only four compounds (linalool, citronellol, *trans*-geraniol, citronellyl acetate) have positive coefficients with PC_1_ in 2013–2014 (**Figure 6C**). On the other hand, highly negative loading values were found with heptadecane (-0.97 and -0.82), octadecane (-0.98 and -0.84), nonadecene (-0.92 and -0.89), nonadecane (-0.97 and -0.97), heneicosane (-0.95 and (-0.94), and tricosane (-0.92 and -0.71) in both the cropping seasons (**Figures 6A,C**).

The PCA bi-plots also indicated that there were three and four distinct groups among the treatment combinations during 2012–2013 and 2013–2014, respectively. The treatment PM_0_ (partial pruning with water spray control) and PM_1_ (partial pruning with foliar application of MgSO_4_ @ 5.0g L^-1^) were separated by the PC_1_ from the rest of the treatments and placed in the negative end of the PC_1_ in 2012–2013 (**Figure 6B**). These two treatments were separated from each other by PC_2_. In 2013–2014, the treatments PM_2_ (partial pruning with foliar application of MgSO_4_ @ 10.0g L^-1^), PM_3_ (partial pruning with foliar application of MgSO_4_ @ 15.0g L^-1^), and CM_4_ (complete pruning with foliar application of MgSO_4_ @ 20.0g L^-1^) were separated from rest of the treatments, and they formed individual group (**Figure 6B**).

## Discussion

The photosynthesis process and synthesis of non-structural carbohydrates are influenced by pruning practices (Chesney and Vasquez, 2007), and the non-structural carbohydrates, which are stored in the pruned plant and used for plant regrowth (Loescher et al., 1990). In this study, new shoot initiation rate (4.63 and 10.80 No. old shoot^-1^) was significantly (*P* ≤ 0.05) higher with partial pruning system compared with the complete system. This result might be due to larger number of dormant vegetative buds present in partially pruned bushes. Moreover, partial pruning maintains a sequence of axes leading from leaves to stem and root system for allocation of photosynthates (Chesney and Nygren, 2002; Chesney, 2008). In our earlier research, higher new shoot initiation rate had also been recorded with top pruning system (Pal et al., 2014). Irrespective of foliar application of MgSO_4_, partial pruning system registered about 22 and 77% higher number of flowers compared with complete pruning system during 2012–2013 and 2013–2014, respectively. These results might be due to the fact that the partial pruning increased light interception within its canopy, maintained adequate amount of metabolic sinks and improved stem water potential.

Though the flower weight (g flower^-1^) was not significantly (*P* ≥ 0.05) affected by pruning system, the partial pruning system produced significantly (*P* ≤ 0.05) higher flower yield compared with complete pruning system, regardless of foliar spray. This result might be attributed to the cumulative effects on higher new shoot initiation rate and number of flower (No. bush^-1^) which ultimately enhanced flower yield. The flower yield is positively correlated with new shoot initiation rate (*r* = 0.74, *P* ≤ 0.05) and number of flower (*r* = 1.00, *P* ≤ 0.01) (**Figure 2**). It had been reported that partial pruning increased relative water content (RWC) and maintained higher photosynthesis process and nutrient supply through root profile (Saifuddin et al., 2010). In our experiment, the variations in flower yield over the years were quite noticeable, and higher yield was recorded in first cropping season (**Table 2**). These results could be due to the fact that the environmental conditions, particularly temperature and rainfall were not favorable during vegetative growth phase and reproductive stage (**Figure 1**). The maximum and minimum temperature (°C) at flower bud formation and flowering stages in 2013–2014 were quite low compared with 2013–2014 cropping season (**Figure 1**). However, the effect of partial pruning system to increase flower yield was more pronounced in second cropping season (**Table 2**). The higher flower yield with partial pruning system might be attributed to the higher level of photosynthetic pigment and N content in leaves (**Table 3**). Though the overall yield in first cropping season was higher, the yield increased by the foliar application of MgSO_4_ @ 15.0g L^-1^ was more consistent over the years.

On the other hand, Mg^2+^ plays a major role in plant photosynthesis and improvement of plant health (Ding et al., 2008; Hermans et al., 2010). Nevertheless, the uptake of Mg^2+^ by plant is lower than K, and its deficiency is a more serious problem in rainfed acidic soil conditions due to the interaction with aluminum (Al). In this study, regardless of pruning system, the maximum number of flower (357.50 and 250.67 no. bush^-1^) and flower yield (1114.47 and 830.69 g bush^-1^) were recorded with the foliar application of MgSO_4_ @ 15.0g L^-1^ during both the cropping seasons. This result may be due to the fact that the foliar application of MgSO_4_ under rainfed conditions increases the availability of Mg for formation of photosynthetic pigment and hastens physiological activities for flower buds formation. The effect of Mg on the various chloroplast enzymes has been reported by Shaul (2002). Furthermore, it had been reported that an adequate supply of Mg increased the activities of antioxidative enzymes and the content of antioxidant molecules in many crops (Cakmak and Marschner, 1992; Cakmak, 1994; Candan and Tarhan, 2003; Tewari et al., 2004, 2006; Anza et al., 2005; Ding et al., 2008; Waraich et al., 2012). We also observed the second degree polynomial relationship (y = 721.31 + 25.707 x - 0.894 x^2^; *R*^2^ = 0.752) between flower yield and level of MgSO_4_ doses (**Figure 2**). In our experiment, total Chl concentration and nitrogen content in the leaves were higher in all MgSO_4_ treated plants (**Table 3**). This result may be a cause to increase the number of flowers and flower yield in MgSO_4_ treated plots in the present study.

Regardless of foliar spray, the Chl content in leaves was considerably higher with the partial pruning system compared with a complete pruning system during both the years. These results could be due to the fact that partial pruning increased light interception within its canopy and hastened cytokinin activities. On the other hand, foliar application of MgSO_4_ also considerably increased the total Chl content in leaves in our study, regardless of pruning system. These results may be due to the fact that the Mg is the central atom of the chlorophyll molecule, hence plays a major role in plant photosynthesis (Ding et al., 2008; Hermans et al., 2010). It has been reported that various chloroplast enzymes are influenced by the minute differences in Mg level (Shaul, 2002). The ribulose-1, 5-bisphosphate (RuBP) carboxylase, a key enzyme in the photosynthesis process, is an important Mg-activated enzyme (Cakmak and Yazici, 2010).

The concentrations of N and K in the leaves were not influenced by pruning system in 2012–2013 cropping season. These results could be due to the dilution effect of nutrient content. However, in 2013–2014, the N, P, and K accumulations in leaves were significantly increased (*P* ≤ 0.05) with partial pruning system (**Table 3**). These results could be due to the fact that partial pruning increased the root proliferation, in which root uptakes more nutrients from larger area of greater depth. The results are in conformity with the findings of Saifuddin et al. (2010). On the other hand, N and P concentrations in leaves were marginally increased with MgSO_4_ treated plants compared with water spray control. These results could be due to the fact that the Mg deficiency prevents uptake of mineral nutrients under rainfed acidic soil conditions. Thus, foliar application of Mg is the effective measure to increase the nutrient uptake pattern in these situations.

The essential oil yield of *R. damascena* is extremely low compared to other essential oil-bearing crops. In our experiment, the average essential oil content in the fresh flower varied from 0.039 to 0.046% depending upon the pruning systems, MgSO_4_ doses, and cropping seasons; however, this variation was not significant (*P* ≥ 0.05). Among the foliar spray, moderate level of MgSO_4_ registered little bit higher oil content. This result may be due to the fact that Mg and its counter ion sulfur influence various biochemical activities. The effects of sulfur to increase the essential oil content have been reported in dragonhead plants (Aziz et al., 2010) and basil (Zheljazkov et al., 2008). It had also been reported that foliar application of Ca and Mg increased oil yield of *Origanum vulgare* (Dordas, 2009). The major components of rose essential oil are citronellol, nerol, geraniol, linalool, methyl eugenol, and hydrocarbon, which decide the perfumery value of rose oil (Lawrence, 1991; Baser, 1992). Though, the least numbers of compounds were identified with PM_0_ (partial pruning system with water spray control), the shearing percentage in total volume was quite high. Thus, the numbers of compounds are not responsible for contributing in total volume of rose essential oil.

In this study, the major components were considerably influenced by the interactive effect of pruning system and foliar application of MgSO_4_ in both the years (**Figure 5**). The accumulation patterns of different volatiles were very dynamics, with citronellol varying from 19.75 to 48.88%, E-geraniol from 9.63 to 29.68%, Z-citral from 0.07 to 5.97%, nonadecane from 6.76 to 22.32, and heneicosane from 2.87 to 10.21%. Two major monoterpenoids citronellol and E-geraniol, attained its maximum level of accumulation with PM_0_ and PM_3_, respectively, in 2012–2013 (**Figure 5C**). However, the minimal accumulation of nonadecane was recorded with CM_1_ followed by PM_2_. These results may be due to the fact that the pruning system coupled with foliar application of MgSO_4_ influences the biosynthesis of various compounds. The influences of foliar application of plant nutrients to change the percentages of major components of essential oils have been reported in many medicinal and aromatic plants such as oregano (Dordas, 2009), French tarragon (Heidari et al., 2014), and lemongrass (Zheljazkov et al., 2011). The multivariate analysis was also conducted by means of PCA for chemical composition. The PCA bi-plots indicated the relation among the variables (**Figures 6A,C**). The analyzed data indicates that the compounds *viz.*, α-pinene, β-pinene, myrcene, linalool, phenyl ethyl acetate, and E-geraniol have highly positive coefficient with PC_1_ in first cropping season. Thus, these compounds are influenced by the similar factors. On the other hand major hydrocarbons such as nonadecene, nonadecane, and heptadecane are positively and highly correlated with each others. Thus the inverse relationship was found between hydrocarbons and monoterpene.

## Conclusion

The results reveal that the pruning system and foliar application of MgSO_4_ alter the flowering behavior, flower and essential oil yield, and profiling of secondary metabolites of *R. damascena* under rainfed acidic conditions. The partial pruning system produced significantly (*P* ≤ 0.05) higher flower yield compared with complete pruning system, regardless of foliar spray. However, the effect of pruning system on flower yield was not consistent over the years. On the other hand, the foliar application of MgSO_4_ @ 15.0g L^-1^ registered about 26–38% higher flower yield compared with water spray control. The effects of partial pruning system and foliar application of MgSO_4_ to increase flower yield were more pronounced in second cropping season. Substantial variations in major compounds (citronellol, E-geraniol, Z-citral, nonadecane, and heneicosane) of essential oil were also observed in this experiment. Thus, it can be concluded that the partial pruning system and foliar application of MgSO_4_ @ 15.0g L^-1^ may be adopted to increase the flower and oil yield with desired quality. However, further studies are required to understand the role of other factors particularly plant nutritions and environmental factors on enzymatic activities.

## Author Contributions

PP: Develop the concept, design the experiment, data analysis, and manuscript writing. MM: Data collection and chemical analysis.

## Conflict of Interest Statement

The authors declare that the research was conducted in the absence of any commercial or financial relationships that could be construed as a potential conflict of interest.
